# Viruses disregulate cell membrane receptors to become immune fugitives

**DOI:** 10.1038/s42003-021-02618-9

**Published:** 2021-09-15

**Authors:** Marissa Knoll

**Affiliations:** Center for Genomics and Systems Biology, New York University, New York, NY USA

## Abstract

In order to maintain persistent infections, microbes that cause chronic disease have to evade detection by the human immune system. To do so, many modulate the expression of plasma membrane receptors that trigger cell signalling pathways and immune responses. Using microscopy and cell sorting techniques, Businger et al. map the morphological changes in the plasma membranes of macrophages infected by human cytomegalovirus or human immunodeficiency virus and find novel differentially expressed receptors.

One of the most significant obstacles any microbe has to overcome in order to launch a successful infection is the plasma membrane, which forms the barrier between the inside of a cell and its environment. In addition to acting as a barrier, the plasma membrane also contains many different molecules and receptors that are important for cell signalling. When faced with infection, some of these receptors are activated and recruit immune cells that attack the invading microbes. For many diseases, this results in an acute infection that is cleared relatively quickly but some microbes are able to avoid detection by the immune system and cause long-lasting, chronic infections. Two examples of this are human cytomegalovirus (HCMV) and human immunodeficiency virus type 1 (HIV-1). Both of these viruses infect cells of the immune system and are known to change the expression of certain surface receptors. However, a comprehensive assessment of how plasma membrane morphology is altered in response to infection has so far been lacking.

In this study, Businger et al.^1^, took advantage of microscopy and cell sorting techniques to assess these infection-induced changes in the plasma membrane. To do so, they infected macrophages with both HCMV and HIV-1 that were labelled with a fluorescent protein, which allowed them to determine if cells became infected or not. Some of the cells that were treated with the virus did not become infected and these were referred to as bystander cells while other cells were left untreated as a negative control (mockinfected). Four days after infection, they then measured the morphology of the macrophages and compared differences between infected, bystander, and mock-infected cells. For both viruses, the height, area, and volume of the cells were unchanged in the infected cells. After infection with HCMV, the macrophages were significantly smoother than either the bystander or mock-infected cells but this change was not observed in the HIV-1 infected cells.

To further assess this change in smoothness, the group tested for changes in plasma membrane receptors. After HIV-1 infection, only a small number of receptors were shown to be differentially regulated, most of which had already been well documented as having a role in HIV-1 biology. However, after HCMV infection, 42 plasma membrane receptors were found to be differentially expressed between infected and bystander cells, 18 downregulated and 24 upregulated, similar to the comparison with mock-infected cells. The group found that the downregulated receptors were mainly involved in regulation of immune responses and activation of other immune cells while the upregulated receptors were involved in inflammatory responses. The screen picked up receptors known to be regulated by infection but also picked up receptors that had not been previously identified in this context. Three of these receptors were found to be strongly downregulated in infected cells: CD164, which is involved in proliferation and migration of immature immune cells, and CD84 and CD180, which are involved in the regulation of immune responses and activation of various immune cell populations. When the group tested a strain of HCMV that had mutations in common immune evasion genes, they found no difference in the expression of these three receptors, suggesting that alternative viral mechanisms may be contributing to immune evasion in HCMV infection. In addition, clinical strains showed the same patterns of expression as lab strains, providing strong evidence that the downregulation of these receptors is important in vivo.

This study documents the morphological changes in response to HCMV and HIV-1 infection and determines that while HIV-1 does not induce large-scale changes in macrophage morphology, HCMV elicits significant changes in plasma membrane morphology. These findings suggest different strategies for persistence between these two viruses as well as identifying new receptors involved in immune evasion during HCMV infections.
